# Nitrogen-fixing symbiotic bacteria act as a global filter for plant establishment on islands

**DOI:** 10.1038/s42003-022-04133-x

**Published:** 2022-11-10

**Authors:** Camille S. Delavaux, Patrick Weigelt, Susan M. Magnoli, Holger Kreft, Thomas W. Crowther, James D. Bever

**Affiliations:** 1 Department of Environmental Systems Science, ETH Zurich, Zurich, Switzerland; 2 Department of Biodiversity, Macroecology & Biogeography, University of Gottingen, Göttingen, Germany; 3 Department of Ecology and Evolutionary Biology, The University of Kansas, Lawrence, KS USA; 4 Kansas Biological Survey, The University of Kansas, Lawrence, KS USA

**Keywords:** Biogeography, Community ecology

## Abstract

Island biogeography has classically focused on abiotic drivers of species distributions. However, recent work has highlighted the importance of mutualistic biotic interactions in structuring island floras. The limited occurrence of specialist pollinators and mycorrhizal fungi have been found to restrict plant colonization on oceanic islands. Another important mutualistic association occurs between nearly 15,000 plant species and nitrogen-fixing (N-fixing) bacteria. Here, we look for evidence that N-fixing bacteria limit establishment of plants that associate with them. Globally, we find that plants associating with N-fixing bacteria are disproportionately underrepresented on islands, with a 22% decline. Further, the probability of N-fixing plants occurring on islands decreases with island isolation and, where present, the proportion of N-fixing plant species decreases with distance for large, but not small islands. These findings suggest that N-fixing bacteria serve as a filter to plant establishment on islands, altering global plant biogeography, with implications for ecosystem development and introduction risks.

## Abstract (web summary)

Plants associated with nitrogen-fixing bacterial symbionts are disproportionately underrepresented on islands, suggesting that these bacteria might play a limiting role in island plant biogeography.

## Introduction

Mutualisms between organisms across the tree of life play a pivotal role in shaping the diversity and functioning of ecosystems. The plant kingdom, in particular, has harnessed symbionts to facilitate plant growth and survival across almost all environments on Earth. Plant mutualisms provide essential services and resources, from pollination to nutrient supply, drought resistance, and pathogen protection^1–5^. One important group of mutualists that associate with plants is nitrogen-fixing (N-fixing) bacteria. In this symbiosis, N-fixing bacteria fix atmospheric nitrogen, with up to 90% used by the plant host^3,6^. These N-fixing mutualisms are major drivers of the global nitrogen cycle, producing up to 100 million tons of terrestrial nitrogen a year^3^, and have large effects on ecosystem development as nitrogen is often limiting in terrestrial systems, particularly on recently formed land such as oceanic islands^7^. While classical island biogeography has historically centered around the spatial and bio-physical drivers of plant distributions, it remains unclear whether co-colonization requirements of the two partners in this mutualism limit the establishment of N-fixing plants on distant islands.

Historically, microbes were assumed to be ‘everywhere’, or have a cosmopolitan distribution, with the environment being the major limiting factors of their realized niche^8^. If this were the case, we would expect plant species associating with mutualists to colonize islands similarly to plant species not associating with these partners, as their colonization would not be limited by this biotic interaction. However, recent work has identified that plant-associated microbes can be dispersal limited, with cascading impacts on their plant hosts^9^. In particular, recent work suggests that biogeographical patterns of plants are consistent with the dispersal limitation of mycorrhizal fungi, particularly arbuscular mycorrhizal (AM) fungi, filtering out AM plant species on islands^10–12^. Whether N-fixing bacteria, the other major plant-microbial symbiont, could similarly limit island colonization by N-fixing plants remains unknown. Such a filter may be expected given that these bacteria can be poor dispersers and can show high specificity for their plant partners^3,13^. Alternatively, N-fixing bacteria could lead to a weak colonization filter as they are able to live saprotrophically^14^, allowing a greater time window between the arrival of the bacteria and the host plant. However, the large plasmid required for symbiosis is costly to maintain, resulting in strong selection against its’ persistence in absence of host plants^15^, again potentially generating a stronger colonization filter of N-fixing plants.

If present, this N-fixing symbiont filter could have important consequences for ecosystem development and diversification patterns. A reduced proportion of N-fixing plant species and their associated symbionts would be expected to contribute to slower development of island ecosystems over time, with slower accumulation of nitrogen through N-fixing bacteria. Thus, an N-fixing symbiont filter on islands could prolong the observed pattern of low nitrogen on younger soils^16–18^ such as those found on oceanic islands. In addition, limited colonization of N-fixing plant species could leave open niches on islands, with consequences for subsequent diversification. Initial filtering out of N-fixing plants may allow subsequent disproportionate diversification of N-fixing compared to non N-fixing plants. Such a pattern of increased diversification resulting in higher endemism for AM plants was observed following a strong colonization filter^12^, but this pattern remains to be tested for N-fixing plants.

Finally, as many plants that associate with N-fixing bacteria also associate with mycorrhizal fungi^17^, it is possible that filters from these two symbionts act non-additively in limiting plant establishment on islands. Synergistic limitation, where plant species that associate with both types of symbionts are less likely to colonize islands than expected from the two filters individually, may occur given that synergistic effects on plant growth has been found for co-inoculation with mycorrhizal fungi and N-fixing bacteria^19–21^. However, meta-analyses suggest that synergistic impacts on plant growth may be limited to perennial plants^22,23^, which may reduce the likelihood of finding synergistic limitations on island colonization. To date, the possibility of a plant island colonization filter strengthened by multiple mutualisms has not been tested.

Here, we use a global database of 209,189 plant species and their N-fixing status^24,25^ across 1005 locations to test for evidence of an N-fixing symbiont filter on plant colonization to islands. We further test if strength of this filter increases with island isolation as dispersal limitation of bacteria is likely to be highest on islands that are more distant from the mainland. In addition, to test if diversification relates to N-fixing status, we explore how these patterns interact with endemism. Finally, we ask if there is a synergistic effect between the mycorrhizal and N-fixing symbiont filter on plant colonization. We test these biogeographical patterns first using all vascular plant species and then within the legume family only. We examine legumes separately because these plants overwhelmingly associate with N-fixing bacteria, have multiple independent losses and gains of associations with mycorrhizal fungi^26^, and are of high economic and agricultural importance^6^. Together, this work tests for the generality of the microbial mutualism filter of plant establishment on islands worldwide. Understanding the impact of mutualisms on island plant establishment has important implications for the field of island biogeography, implicating biotic interactions as a pervasive control on plant distributions.

## Results and discussion

We find evidence for mutualistic limitation of plant establishment by N-fixing bacteria. Across all plant species, N-fixing plants are underrepresented by 22% on both non-oceanic and oceanic islands, consistent with the hypothesis of absence of N-fixing bacterial symbionts on islands limiting host plant establishment. We further find that the probability of N-fixing plant occurrence declines with distance from mainland, and, where present, the proportion of N-fixing plant species declines with distance for all but the smallest islands. Thus, despite tens of millions of years over which islands may accumulate N-fixing species, we detect evidence consistent with an N-fixing symbiont filter on plant establishment on islands worldwide.

### N-fixing plant species are underrepresented on islands

**Fig. 1:**
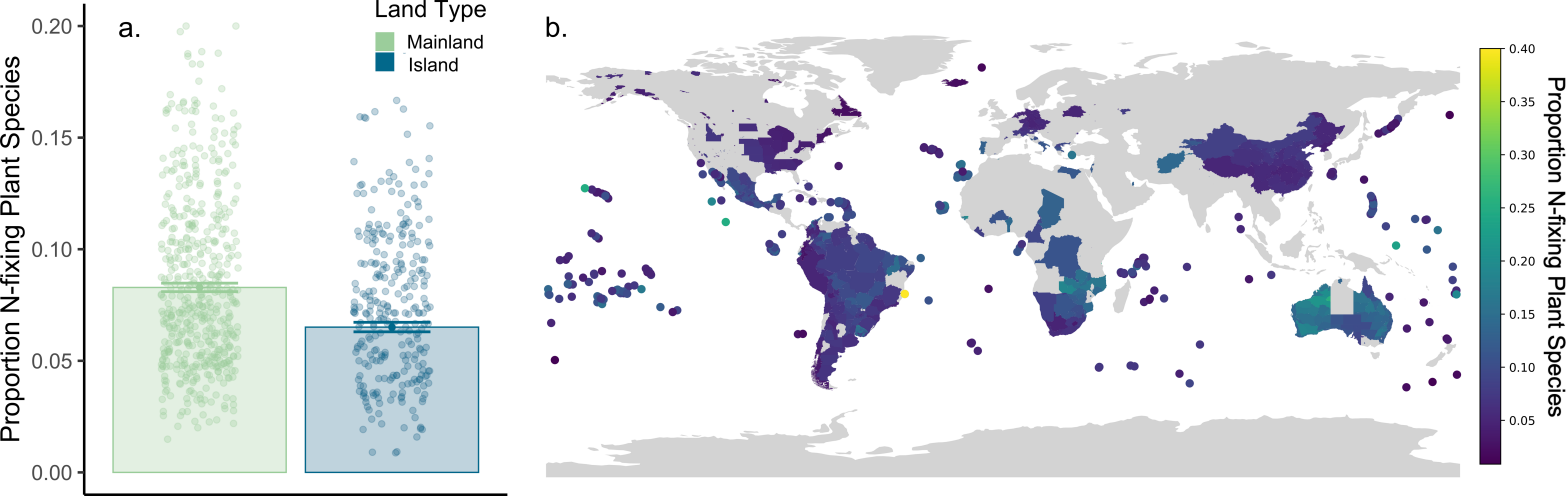
N-fixing plant species are underrepresented on oceanic islands. The proportion of N-fixing plant species is significantly lower on oceanic islands compared to mainlands for all vascular plant species (GLM, **a**, *p* < 0.001, *N* = 1005). Map of proportion N-fixing plant species at locations included in the study (**b**), with dark blue indicating lowest, and yellow indicating highest, proportion N-fixing plant species. Error bars represent standard errors.

N-fixing plant species were underrepresented on islands of both non-oceanic and oceanic origin across all vascular plant species. Specifically, there was a decrease in the proportion of N-fixing plant species on oceanic islands compared to mainlands, representing a 21.5% decline in N-fixing plant species (Fig. 1a, b, *p* < 0.001, Supplementary Table 1, Supplementary Fig. 1a). Across the subset of legumes, although not statistically significant (*p* = 0.11, Supplementary Table 6, Supplementary Fig. 1b), we found that N-fixing plant species are underrepresented on oceanic islands, with an estimated 10.8% decline in the proportion of N-fixing legume plant species. Together, these results support the hypothesis that N-fixing bacteria limit plant establishment on islands, altering the biogeography of these islands relative to their mainland counterparts.

### N-fixing symbiont filter strengthens with distance

**Fig. 2:**
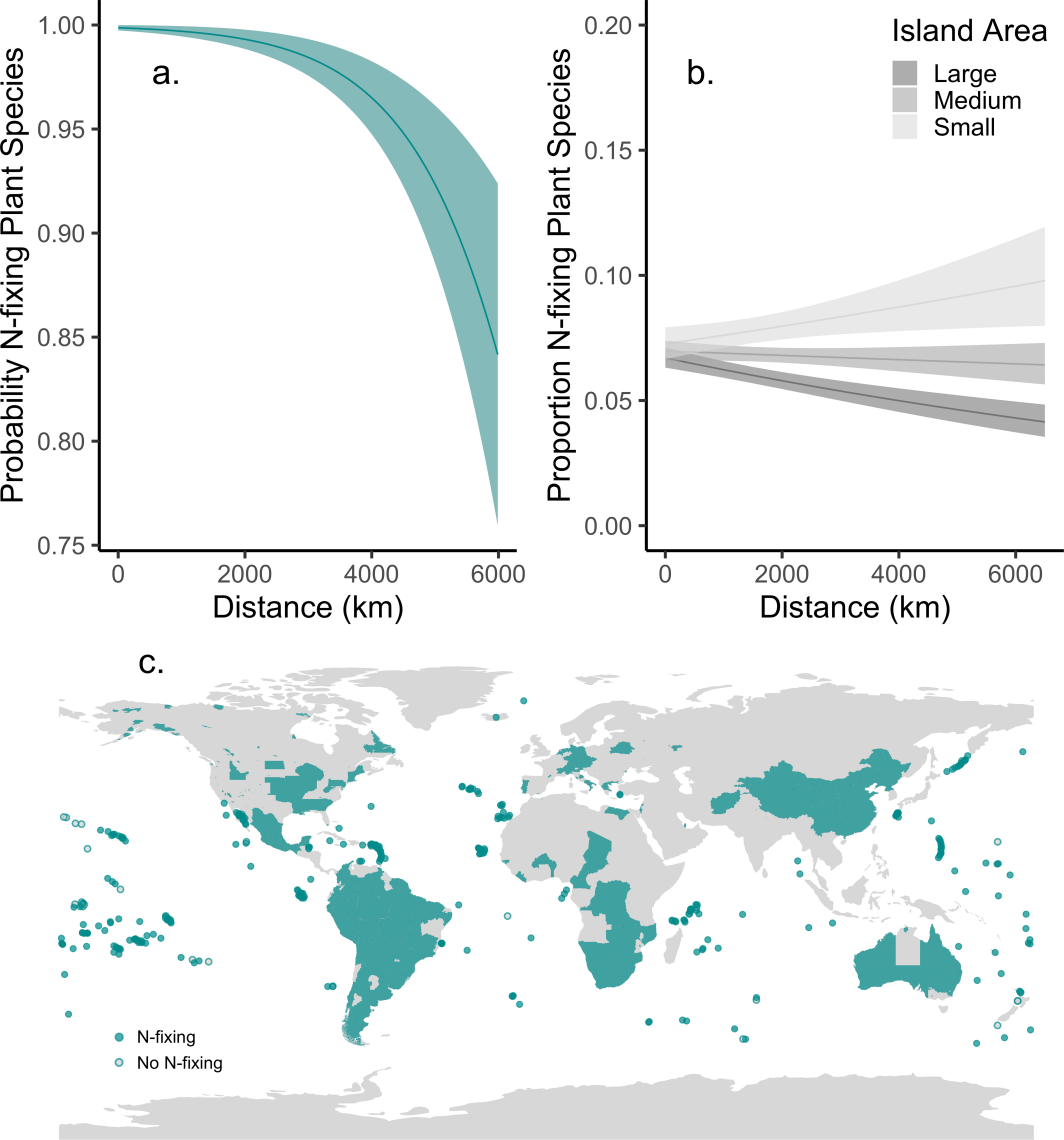
The probability of N-fixing plant species occurrence on oceanic islands decreases with island isolation, while the proportion depends on island area. The probability that N-fixing plant species arrive to oceanic islands decreases with distance from the nearest mainland for all vascular species (GLM, **a**, *p* < 0.001, *N* = 326). The proportion of N-fixing plant species on these islands decreases with distance from mainland only for large, but not small islands (GLM, **b**, *p* < 0.001, *N* = 288; lines represent small (mean − 1 SD), medium (mean) and large (mean + 1 SD) islands). Confidence bands represent 95% confidence intervals. Map of presence of N-fixing plant species globally (C), with filled circles indicating locations with N-fixing plant species present, while empty circles those without N-fixing plant species.

We find evidence for a strengthening of the N-fixing symbiont filter with distance. Across all species, we find that the probability of N-fixing plant species being present on oceanic islands declines with distance from the mainland (Fig. 2a, *p* < 0.001, Supplementary Table 2). Further, when present, we find that the proportion of N-fixing plant species declines with distance from the mainland for large and medium oceanic islands, but not for small islands (Fig. 2b, *p* < 0.001, Supplementary Table 3). The proportion of N-fixing plant species declines with distance for larger islands. On small islands, the increasing proportion of N-fixing plant species with distance may be due to the limited habitat diversity on these islands, with the resulting floras dominated by beach or strand-specialists, a group enriched in N-fixing species. These shared beach colonists across all islands could overwhelm any signal of dispersal limitation by the remaining flora of small islands. Indeed, we find that the shared colonists on small islands show a relatively high N-fixing proportion (8.9%), with these N-fixing plant species found to be widely distributed beach colonists^27–30^. Within the legumes, we find no significant effect of distance on proportion of N-fixing plant species (*p* = 0.58, Supplementary Table 7). This weaker N-fixing symbiont filter effect within the legumes may be due to shared life history traits within legumes, which facilitate their repeated efficient dispersal, ultimately weakening the N-fixing symbiont filter. Alternatively, differential diversification or extinction within this group could explain this weaker observed filter in legumes. Overall, these results provide evidence of a strengthening of the N-fixing symbiont filter with distance, consistent with dispersal limitation of N-fixing symbionts limiting establishment of plant host species.

### Mutualist co-limitation of plant establishment

Plant species that experience both an N-fixing and mycorrhizal filter may be even more strongly underrepresented on islands compared to those that only employ one of these two microbial symbioses. In addition, the fact that many N-fixing plants also associate with mycorrhizal fungi raises the possibility that the mycorrhizal filter may confound the estimates of the filter due to availability of N-fixing bacteria. We tested the possibility of co-limitation (synergism) by mycorrhizal fungi and N-fixing bacteria on plant establishment. We found that both filters act independently, with no evidence that plants that associate with both symbiont types are more strongly limited than expected from the two filters (*p* = 0.20, Supplementary Table 4, Supplementary Fig. 2). This lack of dependency supports the independent operation of both the mycorrhizal and N-fixing symbiont filters.

### Endemism patterns do not support greater diversification of N-fixing plant species with distance

We do not find evidence for differential diversification of N-fixing species with distance from the mainland (all species *p* = 0.27, Supplementary Table 5; legume *p* = 0.46, Supplementary Table 8). This is in contrast to previous work showing greater diversification with distance for AM plant species that experienced an initial underrepresentation at more distant islands. However, further work should test for this pattern, as our sample size for endemism was relatively low (169 islands). We do find that island area is an important predictor of endemism, with the proportion endemic plant species increasing with area (Supplementary Fig. 3, *p* < 0.001, Supplementary Table 5) and that this effect of area is strong for N-fixing plants for both the all species and legume analyses (Supplementary Fig. 3a, b, *p* < 0.001). This may be the result of greater heterogeneity on larger islands providing greater niche space, in turn supporting the diversification of more diverse plant assemblages.

Here, we present results consistent with N-fixing bacteria limiting establishment of N-fixing plant species on islands. We find that the proportion of N-fixing plant species is lower on oceanic islands compared to mainlands. Further, the probability of N-fixing plant species presence declines with island distance, and, where present, the proportion of N-fixing plant species declines with distance for large, but not small islands. While many factors influence island colonization, these patterns of N-fixing plant species are unlikely to result from factors other than a symbiont filter, as young oceanic islands are typically N-limited^16–18,31,32^. Moreover, N-fixing plants are spread across a large portion of the angiosperms^24^; therefore, the operation of an alternative shared trait in driving this pattern is unlikely. In addition, the consistency of observed patterns between the analyses including all angiosperms and those only within legumes suggests patterns are not driven by a legume specific trait. Our results suggest that limited dispersal of N-fixing bacteria can limit the establishment of plants that associate with them, an interpretation supported by findings that N-fixing plants are less successful invaders on mainland systems^13^. Future work should expand on the findings presented here, for example by conducting higher resolution field sampling to determine N-fixing status in situ and relative proportion of this status within the flora as well as through experimental tests of plant colonization with and without N-fixing bacteria.

The presence of an N-fixing symbiont filter on plant species establishment on islands could have consequences for ecosystem development and subsequent invasion risks. Specifically, our results identify that the long-observed pattern of nitrogen limitation on younger soils, particularly on islands^16–18,31,32^, may be made more acute for distant oceanic islands because of limited symbiont dispersal. The N-fixing symbiont filter then, could slow nitrogen accumulation on islands, thereby slowing ecosystem development. Moreover, this initial biotic filter and slower nitrogen accumulation could make islands particularly vulnerable to human-mediated introduction by N-fixing plants and their symbionts, which have been shown to be particularly problematic on islands^33–35^. These island-specific factors could lead to invasion meltdown by N-fixing plant species^36^, dramatically altering these native ecosystems. Therefore, improved conservation and invasion risk mitigation demands that future work determine the impact of introduced plant species on these biogeographical patterns.

The patterns we uncover here, together with previous work showing mycorrhizal fungal limitation of island plant establishment^10,12^, provide evidence for the operation of a general mutualist filter on island plant establishment and biogeography. We show that N-fixing plant species are strongly underrepresented on islands, with a 22% decline in the proportion of N-fixing plant species on islands compared to mainlands. Further, the probability of N-fixers establishing on islands decreases with distance as does the proportion of N-fixing plant species for larger islands. Combined with previous work, both mycorrhizal and N-fixing mutualisms, the two most important plant-microbial mutualisms on earth, limit plant establishment on islands, influencing the distribution of plant species worldwide. This highlights the need to integrate the role of biotic, particularly microbial, mutualistic interactions into island biogeography studies and theory, which will enable improved conservation and restoration of island systems.

## Methods

### Plant distribution data, N-fixing status, mycorrhizal status and explanatory variables

Plant species occurrence data (for mostly administratively defined locations such as countries and provinces or islands), biogeographic status (native, endemic) and explanatory variables with characteristics were extracted from the Global Inventory of Floras and Traits v 1.0, GIFT database^25^. We used all locations for which checklists of native angiosperms were available. Endemic plant species are defined as plant species that are unique to a location’s flora in a given checklists. When there were overlapping lists for a location, the smaller locations were kept if larger than 100 km^2^ for mainland locations; for islands, the smaller units were always preferred. Finally, we removed islands for which island geology (i.e., volcanic, floor, shelf, fragment, etc.) was undetermined.

The nitrogen-fixing (N-fixing) status of plant species included in this study was determined by assigning each species to its plant family according to The Plant List^37^, incorporating the classification from APG IV^38^. We used the N-fixing database of 9156 angiosperm species previously published by Werner et al.^24^, which identifies plant species that form nodules to house N-fixing bacteria (i.e., legumes that associate with rhizobia and actinorhizal plant species). We first assigned N-fixing status (N-fixer or non N-fixer) based on species level status in the database. If the given species was not found, we then applied family-level proportions of plant species’ N-fixing status to assign status proportions to the locations’ plant species assemblages. Where neither species nor family was found in the database, species were removed from analyses. In total, we assigned 6,860 species using the N-fixing status database matching directly to species and an additional 202,329 species through family status proportions. We estimate the total number of N-fixing species in our dataset, and the most up to date estimate of N-fixing plant species globally, to be 14,553. Mycorrhizal status was assigned using the FungalRoot database^39^ using the same approach as for N-fixing assignment. That is, we first assigned mycorrhizal status based on species level status; if this was not possible, we applied genus-level proportions to plant species assemblages. Although there are several mycorrhizal types, we were primarily interested in the arbuscular mycorrhizal plant species, as these were previously shown to be underrepresented on islands^12^ and they represent the most common mycorrhizal type. Therefore, we only compared two mycorrhizal statuses: arbuscular mycorrhizal (AM) and all other mycorrhizal types, including non-mycorrhizal plant species (NM).

Explanatory variables for each location were extracted from the GIFT database. For details of environmental data collection, see Weigelt et al.^40^. Explanatory variables included land type (mainland, non-oceanic island, oceanic island), absolute latitude and longitude of the location’s centroid, area (km²), mean annual temperature (°C) and mean annual precipitation^41^ (mm), and elevational range^42^ (difference between lowest and highest elevation in m). When elevation range was unknown or reported as zero from aerial elevation maps, we assigned an elevation of 1 m as a minimum necessary elevation. For islands, we also included island distance to the nearest mainland (km) as a measure of geographical isolation^43^ and island geological age. It is important to note that age reflects geological age, and not biological age, and therefore may not accurately reflect time from start of plant colonization. Data for endemism analyses represent a subset of islands for which endemism status was known.

### Statistics and reproducibility

To investigate patterns of native N-fixing plant distributions globally, we used several mixed models that correct for non-independence due to spatial proximity. First, for all native angiosperm species, and then legumes separately, we tested for overall differences in the proportion of N-fixing plant species between mainlands and islands. We were unable to test for presence in these models as N-fixing plant species were present on all mainlands. Next, for island floras, we tested for environmental drivers of the presence, with sites with a minimum of one N-fixing plant species labeled ‘present’, and proportion of N-fixing plant species for sites with N-fixing plant species present. We then tested for synergism of the N-fixing and mycorrhizal filters. Finally, we reran the island models to test for the proportion of N-fixing plant species among endemics.

In our first set of models, we compared N-fixing plant species to non N-fixing plant species between land types (mainland or island). We used a composite response variable with species richness of N-fixing to non N-fixing plant species to account for differences in species richness. For these analyses, we used generalized linear models (GLMs) with a logit link function, assuming a binomial distribution of the response variable. The fixed effects were land type, absolute latitude, squared latitude, the natural logarithm of area, the natural logarithm of elevation, mean annual temperature and mean annual precipitation. We also included the random effect of location nested within land type. Random terms control for the non-independence of individual plant species records within floras, thereby providing general tests for differences in the proportion of N-fixing plant species across the floras of the different land types.

Next, to investigate geographical and environmental drivers of N-fixing presence and proportion for native plants in oceanic island floras, we ran models comparing either the presence or proportion of N-fixing to non N-fixing plant species. For presence, the response variable was coded as a binary response variable, either the location had at least one N-fixing plant species (present), or it did not (not present). For proportion, we again used a composite response variable with species richness of N-fixing to non N-fixing plant species to account for differences in species richness. For these analyses, we used generalized linear models (GLMs) with a logit link function, assuming a quasi-binomial distribution of the response variable. We included the following explanatory variables: absolute latitude, squared latitude, the natural logarithm of area, the natural logarithm of elevation, mean annual temperature, mean annual precipitation and distance to mainland. We initially explored models with island age, however, as (1) this effect was not significant, (2) inclusion of this predictor substantially reduced the number of location s in the model, and (3) when testing for the best models, age was not included in all but one resulting model, we include results of models without island age in the manuscript. The choice of variables was informed by prior studies of their effects on this dataset^10,12,44^ as well as other island biogeographic studies^45,46^.

To test for synergism between N-fixing and mycorrhizal limitation, we predicted species counts using generalized linear models (GLMs). As covariates, we included N-fixing status (N-fixing or non N-fixing), mycorrhizal status (AM or NM), location, as well as the three-way interaction of these three covariates. A significant three-way interaction between N-fixing status, mycorrhizal status and land type would indicate evidence of synergistic limitation of plant establishment, with the counts of species associating with both symbionts being lower than that predicted from average filter effects of each symbiont individually. The random effects were location nested within land type, the interaction of location nested within land type with N-fixing status, the interaction of location nested within land type with mycorrhizal status, and the interaction of location nested within land type with N-fixing status and mycorrhizal status.

Finally, we ran models testing for the proportion of endemic species in native oceanic island floras, using a composite response variable with counts of endemic species and non-endemic species. For these analyses, we used generalized linear models (GLMs) with a logit link function, assuming a quasi-binomial distribution of the response variable. As covariates, we included N-fixing status, absolute latitude, squared latitude, the natural logarithm of area, the natural logarithm of elevation, mean annual temperature, mean annual precipitation, and distance to mainland. We also tested for the interaction of N-fixing status with area and again with distance.

Where we detected overdispersion (Supplementary Tables 2–5, 7, 8), this was adequately corrected using a quasi-binomial or quasi-Poisson family model. Most of our model residuals showed spatial autocorrelation as tested using Moran’s I, which is expected in global scale models with spatially clustered geographic locations. We corrected for this spatial autocorrelation by including a spatial autocovariate that incorporates a matrix of longitude and latitude coordinates of the locations^46^ in the *spdep* package in R^47^. Neighborhood distance were set to 1000 km and weighted by inverse distance; spatial coordinates are the site centroids, except for endemism models where we lightly staggered centroid locations due to paired rows per site. All analyses were done in R 3.4.1^48^ in the *lme4* package^49^.

### Reporting summary

Further information on research design is available in the Nature Research Reporting Summary linked to this article.

## Supplementary information

Supplementary Information

Description Of Additional Supplementary Files

Supplementary Data 1

Reporting Summary

## Data Availability

The Global Inventory of Floras and Traits (GIFT) database is available at: http://gift.uni-goettingen.de/, with additional geographic information shared upon request. N-fixing status data is from Werner et al.^24^ while mycorrhizal status data is from the FungalRoot database from Soudzilovskaia et al.^39^. All Source Data for each figure are presented in Supplementary Data 1.

## Code availability

All code used in this paper can be found at https://github.com/c383d893/N-fixing_Filter Filter (10.5281/zenodo.7220005).
