# Supplementary material for: Nitrogen-fixing symbiotic bacteria act as a global filter for plant establishment on islands
Source: Commun Biol. 2022 Nov 10;5:1209. doi: 10.1038/s42003-022-04133-x (PMC9649727; doi:10.1038/s42003-022-04133-x)
Supplement: Supplementary file 1 — Supplementary Information [file 42003_2022_4133_MOESM1_ESM.pdf]

Supplementary Information for

Nitrogen-fixing symbiotic bacteria act as a global filter for plant establishment on islands

Supplementary Table 1. N-fixing plant species proportion by land type.

Model results of GLMs conducted with angiosperm species in the dataset, showing the model results from the broad model testing whether proportion N-fixing plant species differs between land type (mainland versus island; N = 1,005). Random effect results are reported below fixed effects.

	Estimate	Std. Error	z -value	p-value
(Intercept)	-2.491	0.023	-108.368	<2.00E-16
landtype (nonoceanic)	-0.219	0.047	-4.675	2.94E-06
landtype (oceanic)	-0.242	0.046	-5.285	1.26E-07
absolute latitude	0.189	0.044	4.345	1.40E-05
squared latitude	-0.097	0.051	-1.899	0.058
area	-0.029	0.028	-1.046	0.296
elevational range	0.022	0.026	0.859	0.390
mean annual precipitation	-0.165	0.015	-10.943	<2.00E-16
mean annual temperature	0.404	0.030	13.318	<2.00E-16
random effect	Variance		Std. Dev.	
land type:location	0.093		0.305	

Supplementary Table 2. Island presence N-fixing plant species.

Model results of GLMs conducted with angiosperm species in the dataset, showing the model results from the island only models, testing drivers of presence of N-fixing plant species (N = 326).

	Estimate	Std. Error	z -value	p-value
(Intercept)	4.928	0.676	7.285	3.21E-13
absolute latitude	1.280	1.021	1.253	0.210
squared latitude	0.598	1.846	0.324	0.746
area	2.098	0.533	3.937	8.26E-05
elevational range	1.355	0.488	2.775	0.006
mean annual precipitation	2.414	0.565	4.272	1.94E-05
mean annual temperature	3.108	1.280	2.428	0.015
distance	-1.492	0.353	-4.227	2.37E-05
spatial autocorrelation variable	-0.050	0.632	-0.079	0.937

Supplementary Table 3. Island proportion N-fixing plant species.

Model results of GLMs conducted with angiosperm species in the dataset, showing the model results from the island only models, testing drivers of proportion of N-fixing plant species (N = 288).

	Estimate	Std. Error	z -value	p-value
(Intercept)	-2.593	0.023	-110.693	<2.00E-16
absolute latitude	0.066	0.065	1.011	0.312
squared latitude	-0.271	0.106	-2.546	0.011
area	-0.170	0.025	-6.755	1.43E-11
elevational range	0.027	0.034	0.806	0.420
mean annual precipitation	-0.110	0.020	-5.631	1.80E-08
mean annual temperature	0.062	0.061	1.026	0.305
distance	-0.025	0.024	-1.033	3.02E-01
area*distance	-0.114	0.022	-5.195	2.05E-07
spatial autocorrelation variable	0.223	0.015	14.920	<2.00E-16

Supplementary Table 4. Co-limitation by mycorrhizal fungi and N-fixing bacteria.

Model results of GLMs conducted with angiosperm species in the dataset, showing the model results testing for co-limitation by mycorrhizal fungi and bacteria (synergism) by testing for a three-way interaction between mycorrhizal status, N-fixing status and land type in predicting species counts (N = 5,117). Random effect results are reported below fixed effects

	Estimate	Std. Error	z -value	p-value
(Intercept)	6.057	0.054	112.638	<2.00E-16
N-fixing (N-fix)	-3.209	0.034	-93.741	<2.00E-16
mycorrhizal (NM)	0.817	0.034	24.234	<2.00E-16
landtype (non-oceanic)	-2.933	0.092	-32.023	<2.00E-16
landtype (oceanic)	-2.961	0.087	-33.930	<2.00E-16
N-fixing*mycorrhizal	1.279	0.038	33.319	<2.00E-16
N-fixing*landtype (non- oceanic)	0.277	0.095	2.921	0.003
N-fixing*landtype (oceanic)	0.021	0.101	0.210	0.834
mycorrhizal*landtype (non- oceanic)	-0.092	0.061	-1.505	0.132
mycorrhizal*landtype (oceanic)	0.179	0.056	3.189	0.001
N-fixing*mycorrhizal*landtype (non-oceanic)	-0.029	0.100	-0.286	0.775
N-fixing*mycorrhizal*landtype (oceainc)	0.137	0.105	1.295	0.195
random effect		Variance		Std. Dev.
land type:location:N-fixing:mycorrhizal		0.173		0.416

land type:location:mycorrhizal	0.118	0.344
land type:location:N-fixing	0.097	0.311
land type:entity_ID	1.097	1.047

Supplementary Table 5. Drivers of proportion endemic plant species on islands.

Model results of GLMs conducted with angiosperm species in the dataset, showing the model results from the island model testing drivers of proportion endemic plant species (N = 169).

	Estimate	Std. Error	z -value	p-value
(Intercept)	-3.071	0.152	-20.208	<2.00E-16
absolute latitude	1.499	0.078	19.211	<2.00E-16
squared latitude	-2.269	0.122	-18.532	<2.00E-16
area	-0.667	0.179	-3.727	0.000
elevational range	-0.200	0.038	-5.304	1.14E-07
mean annual precipitation	0.299	0.025	12.086	<2.00E-16
mean annual temperature	-1.113	0.058	-19.193	<2.00E-16
distance	-0.004	0.151	-0.028	9.78E-01
nitrogen fixing status	0.785	0.154	5.103	3.34E-07
nitrogen fixing status*distance	0.169	0.152	1.113	0.266
nitrogen fixing status*area	1.264	0.179	7.054	1.74E-12
spatial autocorrelation variable	0.236	0.005	50.350	<2.00E-16

Supplementary Table 6. N-fixing legume species proportion by land type.

Model results of GLM conducted with only legume species in the dataset, showing the model results from the broad model testing whether proportion N-fixing legume species differs between land type (N = 807). Random effect results are reported below fixed effects.

	Estimate	Std. Error	z-value	p-value
(Intercept)	2.331	0.028	82.838	<2.00E-16
landtype (nonoceanic)	0.232	0.075	3.106	0.002
landtype (oceanic)	-0.114	0.072	-1.575	0.115
absolute latitude	-0.051	0.067	-0.753	0.451
squared latitude	0.662	0.088	7.523	5.36E-14
area	-0.052	0.032	-1.609	0.108
elevational range	0.206	0.029	7.109	1.17E-12
mean annual precipitation	-0.098	0.021	-4.649	3.33E-06
mean annual temperature	0.164	0.040	4.067	4.76E-05
random effect		Variance		Std. Dev.

land type:location	0.032	0.178

Supplementary Table 7. Island proportion N-fixing legume species.

Model results of GLM conducted with only legume species in the dataset, showing the model results from the island only models, testing drivers of proportion of N-fixing legume species (N = 200).

	Estimate	Std. Error	z-value	p-value
(Intercept)	1.793	0.058	30.919	<2.00E-16
absolute latitude	0.440	0.226	1.950	0.051
squared latitude	-0.425	0.378	-1.125	0.261
area	0.103	0.065	1.579	0.114
elevational range	0.193	0.079	2.452	0.014
mean annual precipitation	-0.040	0.058	-0.679	0.497
mean annual temperature	-0.046	0.199	-0.230	0.818
distance	-0.035	0.063	-0.558	0.577
spatial autocorrelation variable	0.679	0.098	6.898	5.28E-12

Supplementary Table 8. Drivers of proportion endemic legume species on islands.

Model results of GLM conducted with only legume species in the dataset, showing the model results from the island model testing drivers of proportion endemic legume species (N =70).

	Estimate	Std. Error	z-value	p-value
(Intercept)	-2.684	0.153	-17.570	<2.00E-16
absolute latitude	1.756	0.978	1.795	0.073
squared latitude	-0.849	1.005	-0.844	0.398
area	0.878	0.153	5.753	8.78E-09
elevational range	0.601	0.191	3.145	0.002
mean annual precipitation	-0.292	0.173	-1.687	0.092
mean annual temperature	0.646	0.350	1.844	0.065
distance	-0.018	0.178	-0.099	0.921
nitrogen fixing status	1.608	0.266	6.035	1.59E-09
nitrogen fixing status*distance	0.187	0.253	0.741	0.459
nitrogen fixing status*area	-0.910	0.237	-3.839	1.24E-04
spatial autocorrelation variable	0.492	0.096	5.097	3.45E-07

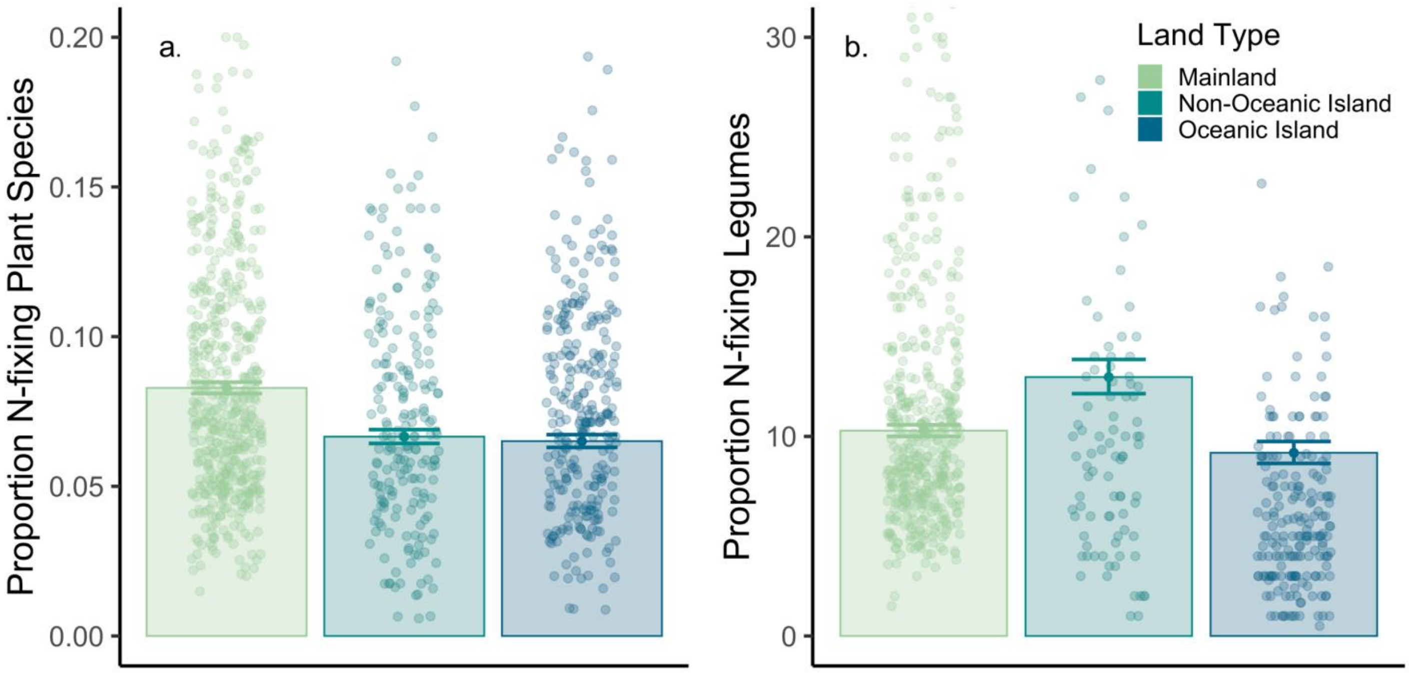

Supplementary Figure 1. N-fixing plant species are underrepresented on islands.

The proportion of N-fixing plant species is significantly lower on islands compared to mainlands for all vascular plant species (a, oceanic and non-oceanic island $p < 0.001$, $N = 1,005$). Within legumes, the proportion N-fixing plant species is higher on non-oceanic islands, and insignificantly lower on oceanic islands (b, non-oceanic $p < 0.001$, oceanic $p = 0.115$, $N = 807$). Error bars represent standard errors.

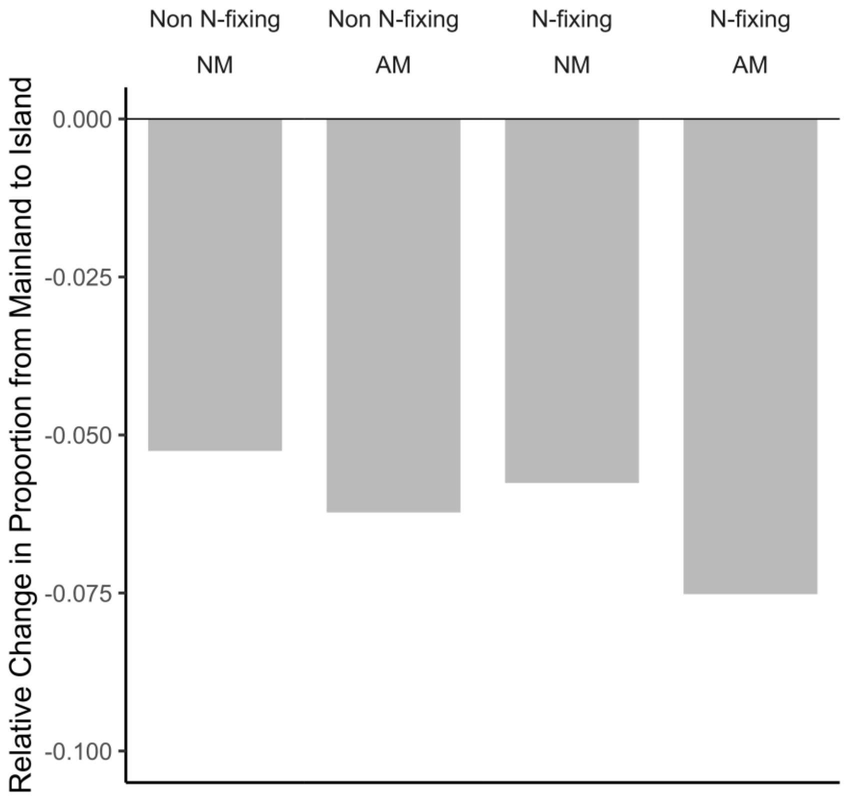

Supplementary Figure 2. Test for synergism in mycorrhizal and n-fixing filter on island plant establishment.

Our test for synergism showed no evidence that plants that associate with both symbiont types are more strongly limited than expected from the two filters ($p = 0.20$, $N = 5{,}117$).

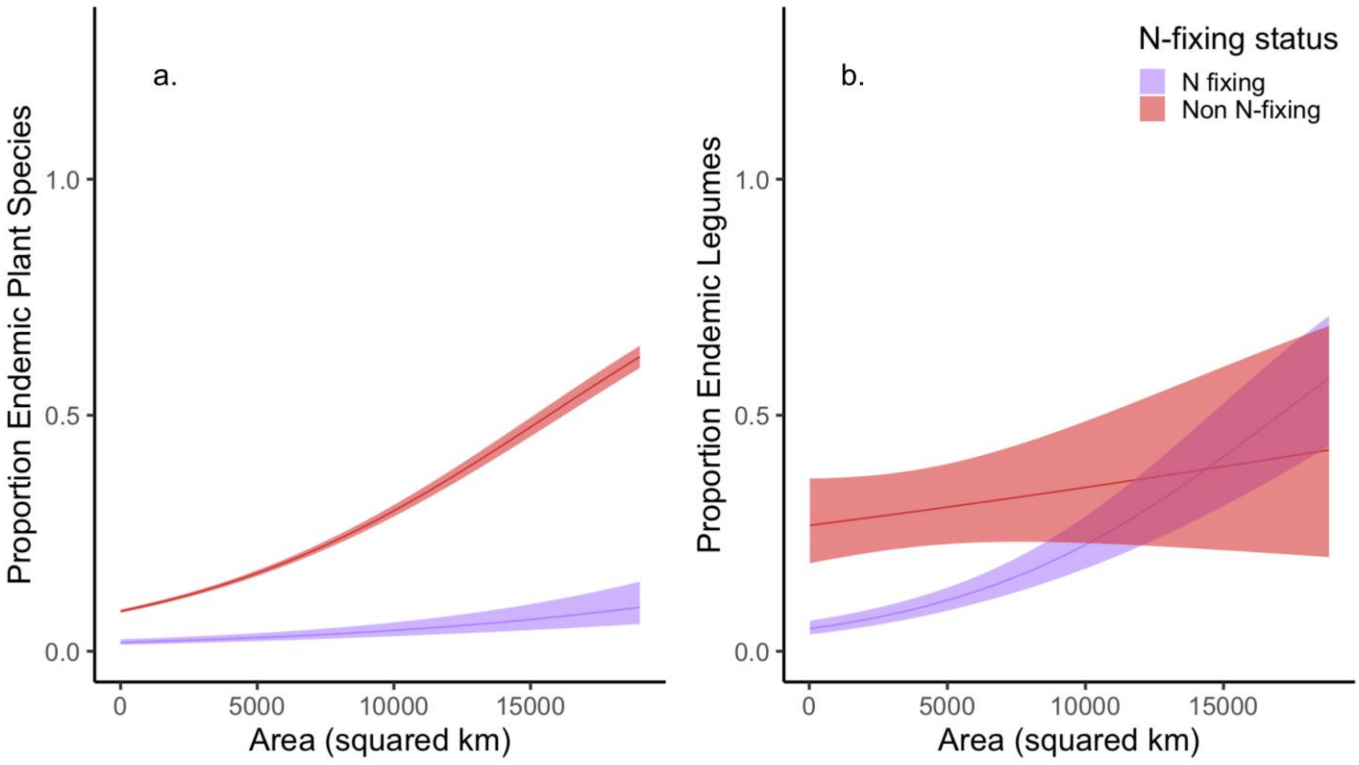

Supplementary Figure 3. Endemism patterns across island area.

For all vascular plant species (a, N = 169), the proportion of endemic plant species increases with increasing island area, with the increase stronger for non N-fixing plant species. For legume plant species (b, N = 170), the proportion endemic plant species increases with increasing island area, with the increase stronger for N-fixing plant species. Confidence bands represent 95% confidence intervals.
