# Supplementary material for: Nitrogen-fixing symbiotic bacteria act as a global filter for plant establishment on islands
Source: Commun Biol. 2022 Nov 10;5:1209. doi: 10.1038/s42003-022-04133-x (PMC9649727; doi:10.1038/s42003-022-04133-x)
Supplement: Supplementary file 2 — Description Of Additional Supplementary Files [file 42003_2022_4133_MOESM2_ESM.pdf]

Description of Additional Supplementary Files

Supplementary Data 1. Source data behind graphs in paper.
This includes data necessary to create all figures in main text (Figures 1 and 2) and Supplementary figures (Supplementary Figures 1-3).
