# Supplementary material for: Nitrogen-fixing symbiotic bacteria act as a global filter for plant establishment on islands
Source: Commun Biol. 2022 Nov 10;5:1209. doi: 10.1038/s42003-022-04133-x (PMC9649727; doi:10.1038/s42003-022-04133-x)
Supplement: Supplementary file 4 — Reporting Summary [file 42003_2022_4133_MOESM4_ESM.pdf]

nature portfolio

Corresponding author(s): Dr. Camille S. Delavaux

Last updated by author(s): 2022.10.09

Reporting Summary

Nature Portfolio wishes to improve the reproducibility of the work that we publish. This form provides structure for consistency and transparency in reporting. For further information on Nature Portfolio policies, see our Editorial Policies and the Editorial Policy Checklist.

Statistics

For all statistical analyses, confirm that the following items are present in the figure legend, table legend, main text, or Methods section.

n/a	Confirmed	
☐	☒	The exact sample size (*n*) for each experimental group/condition, given as a discrete number and unit of measurement
☐	☒	A statement on whether measurements were taken from distinct samples or whether the same sample was measured repeatedly
☐	☒	The statistical test(s) used AND whether they are one- or two-sided<br>*Only common tests should be described solely by name; describe more complex techniques in the Methods section.*
☐	☒	A description of all covariates tested
☐	☒	A description of any assumptions or corrections, such as tests of normality and adjustment for multiple comparisons
☐	☒	A full description of the statistical parameters including central tendency (e.g. means) or other basic estimates (e.g. regression coefficient) AND variation (e.g. standard deviation) or associated estimates of uncertainty (e.g. confidence intervals)
☐	☒	For null hypothesis testing, the test statistic (e.g. *F*, *t*, *r*) with confidence intervals, effect sizes, degrees of freedom and *P* value noted<br>*Give P values as exact values whenever suitable.*
☒	☐	For Bayesian analysis, information on the choice of priors and Markov chain Monte Carlo settings
☐	☒	For hierarchical and complex designs, identification of the appropriate level for tests and full reporting of outcomes
☐	☒	Estimates of effect sizes (e.g. Cohen's *d*, Pearson's *r*), indicating how they were calculated

Our web collection on statistics for biologists contains articles on many of the points above.

Software and code

Policy information about availability of computer code

Data collection	The Global Inventory of Floras and Traits (GIFT) database is available at: http://gift.uni-goettingen.de/, with additional geographic information shared upon request. N-fixing status data is from Werner et al. 2014, while mycorrhizal status data is from the FungalRoot database from Soudzilovskaia et al. 2022 (cited in the text).
Data analysis	All analyses were done in R 4.4.1.

For manuscripts utilizing custom algorithms or software that are central to the research but not yet described in published literature, software must be made available to editors and reviewers. We strongly encourage code deposition in a community repository (e.g. GitHub). See the Nature Portfolio guidelines for submitting code & software for further information.

Data

Policy information about availability of data

All manuscripts must include a data availability statement. This statement should provide the following information, where applicable:

- Accession codes, unique identifiers, or web links for publicly available datasets
- A description of any restrictions on data availability
- For clinical datasets or third party data, please ensure that the statement adheres to our policy

The Global Inventory of Floras and Traits (GIFT) database is available at: http://gift.uni-goettingen.de/, with additional geographic information shared upon request. N-fixing status data is from Werner et al. 2014, while mycorrhizal status data is from the FungalRoot database from Soudzilovskaia et al. 2022 (cited in the text).

Field-specific reporting

Please select the one below that is the best fit for your research. If you are not sure, read the appropriate sections before making your selection.

☒ Life sciences ☐ Behavioural & social sciences ☐ Ecological, evolutionary & environmental sciences

For a reference copy of the document with all sections, see nature.com/documents/nr-reporting-summary-flat.pdf

Life sciences study design

All studies must disclose on these points even when the disclosure is negative.

Sample size	Sample size was determined by including all data with parameters used in any given model.
Data exclusions	From the GIFT database, we only used regions for which checklists for native angiosperms were available. When there were overlapping entities, the smaller regions were kept if greater than 1 km2, otherwise, larger regions were kept. We also removed islands for which geology was undetermined. These were pre-determined exclusion criteria. Please find more details about these exclusions and subsets in the 'Plant distribution data, N-fixing status, mycorrhizal status and explanatory variables' subsection of the Methods section.
Replication	Because of the large (global) scale of this data, we cannot replicate the data collection and subsequent work. Please see more detail in the 'Statistics and Reproducibility' subsection of the Methods section.
Randomization	All detailed data collection can be found in references cited within text and in our data availability section.
Blinding	Investigators were not blinded in this study since the data had already been collected.

Reporting for specific materials, systems and methods

We require information from authors about some types of materials, experimental systems and methods used in many studies. Here, indicate whether each material, system or method listed is relevant to your study. If you are not sure if a list item applies to your research, read the appropriate section before selecting a response.

Materials & experimental systems

n/a	Involved in the study	
☒	☐	Antibodies
☒	☐	Eukaryotic cell lines
☒	☐	Palaeontology and archaeology
☒	☐	Animals and other organisms
☒	☐	Human research participants
☒	☐	Clinical data
☒	☐	Dual use research of concern

Methods

n/a	Involved in the study	
☒	☐	ChIP-seq
☒	☐	Flow cytometry
☒	☐	MRI-based neuroimaging
